# Supplementary material for: Comparative Analysis of the Alkaline Proteolytic Enzymes of Yarrowia Clade Species and Their Putative Applications
Source: Int J Mol Sci. 2023 Mar 30;24(7):6514. doi: 10.3390/ijms24076514 (PMC10095220; doi:10.3390/ijms24076514)
Supplement: Supplementary file 1 [file ijms-24-06514-s001.zip › ijms-2249011-supplementary.pdf]

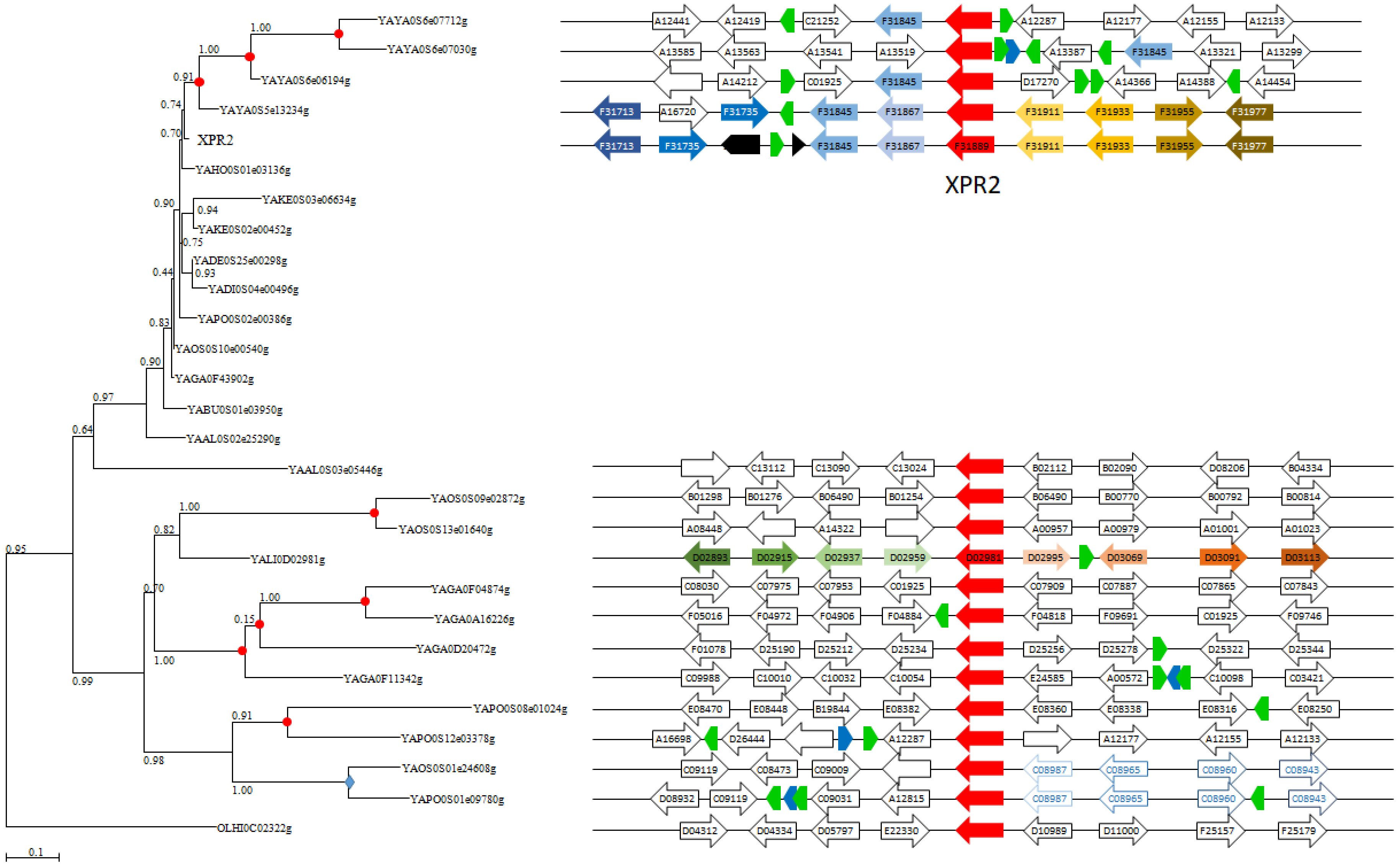

Figure S1. Synteny analysis of alkaline proteases, closely related to *XPR2* homologues emanating from successive duplication events in *Yarrowia* clade species. The phylogenetic tree was constructed from an alignment of 228 amino acids with phyML and a LG evolutionary model. *XPR2* orthologues are in red and flanking genes are in different blue or yellow to brown colours reflecting orthologues in the different species, as in Figure 1. Genes with no homology to each other are represented by white arrows and the name of their homologue in *Y. lipolytica* is written inside. A single exception concerns blue genes inside blue arrows in *Y. osloensis* and *Y. porcina* (see the blue diamond at the ancestral node). Red dots indicate species specific duplications.

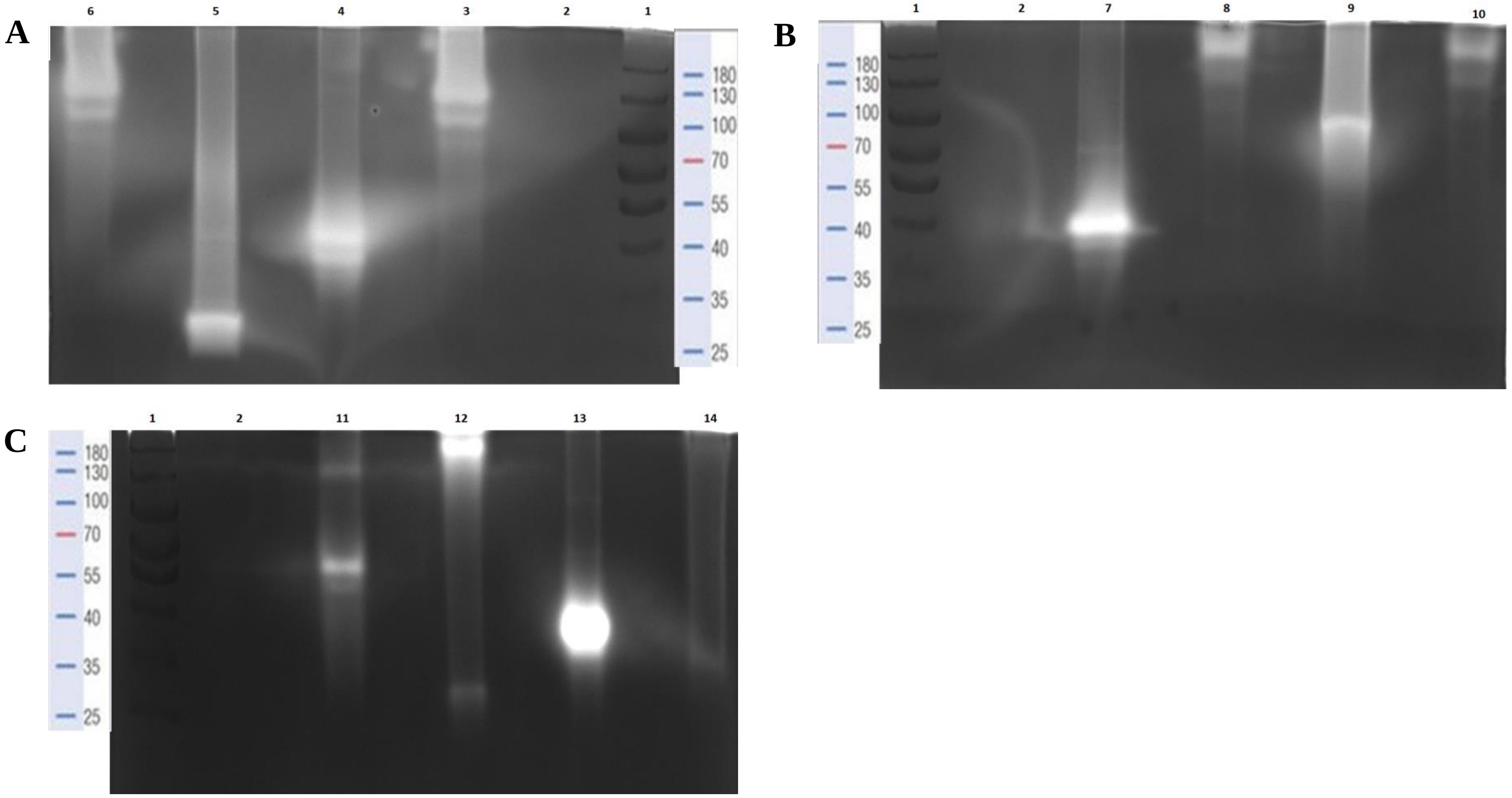

Figure S2. Gelatin zymogram of *Yarrowia* clade's proteolytic enzymes performed on 10% SDS-PAGE. (A) (Lane 1) Molecular mass marker, (lane 2) Control, (lane 3) *Y. lipolytica*, (lane 4) *Y. galli*, (lane 5) *Y. keelungensis*, (lane 6) *Y. alimentaria*. (B) (Lane 1) Molecular mass marker, (lane 2) Control, (lane 7) *Y. parophonii*, (lane 8) *Y. brassicae*, (lane 9) *Y. bubula*, (lane 10) *Y. divulgata*. (C) (Lane 1) Molecular mass marker, (lane 2) Control, (lane 11) *Y. hollandica*, (lane 12) *Y. porcina*, (lane 13) *Y. osloensis*, (lane 14) *Y. yakushimensis*.

Table S1. Results of comparative proteomic analysis of *Yarrowia* clade proteolytic enzymes secreted in the BSG medium.

No.	*Host*	Accession number	Gene name	Protein name	Size observed on gelatin gel	Taxonomy	Protein sequence coverage [%]	Matched peptide sequence	Peptide location Start-End Point
1.	*Yarrowia lipolytica* W29	P09230	YALI0F31889g (XPR2)	Alkaline extracellular protease (AEP)	130	*Yarrowia lipolytica* CLIB122	40	AIQTTPVTQWGLSR	158 – 171
								KAQTGNYAYVR	176 – 186
								AQTGNYAYVR	177 – 186
								HPTVSYVVDSGIR	192 – 204
								TTHSEFGGR	205 – 213
								AVWGANFADTQNADLLGHGTHVAGTVGGK	214 – 242
								TYGVDANTK	243 – 251
								TYGVDANTKLVAVK	243 – 256
								LVAVKVFAGR	252 – 261
								SAALSVINQGFTWALNDYISK	262 – 282
								GVLNFSGGGPK	289 – 299
								GVLNFSGGGPKSASQDALWSR	289 – 309
								SASQDALWSR	300 – 309
								ATQEGLLVAIAAGNDAVDACNDSPGNIGGSTSGIITVGSIDSSDK	310 – 354
2.	*Yarrowia lipolytica* W29	P09230	YALI0F31889g (XPR2)	Alkaline extracellular protease (AEP)	110	*Yarrowia lipolytica* CLIB122	44	AIQTTPVTQWGLSR	158 – 171
								KAQTGNYAYVR	176 – 186
								AQTGNYAYVR	177 – 186
								ETVGKHPTVSYVVDSGIR	187 – 204
								HPTVSYVVDSGIR	192 – 204
								TTHSEFGGR	205 – 213
								AVWGANFADTQNADLLGHGTHVAGTVGGK	214 – 242
								TYGVDANTK	243 – 251
								TYGVDANTKLVAVK	243 – 256
								LVAVKVFAGR	252 – 261
								SAALSVINQGFTWALNDYISK	262 – 282
								GVLNFSGGGPK	289 – 299
								SASQDALWSR	300 – 309
								ATQEGLLVAIAAGNDAVDACNDSPGNIGGSTSGIITVGSIDSSDK	310 – 354
								GSPNAVAYNGVGI	442 – 454
3.	*Yarrowia keelungensis*	P09230	YALI0F31889g (XPR2)	Alkaline extracellular	30	*Yarrowia lipolytica*	35	AIQTTPVTQWGLSR	158 – 171
								AQTGNYAYVR	177 – 186

	CBS 11062			protease (AEP)		CLIB122		HPTVSYVVDSGIR	192 – 204
								TTHSEFGGR	205 – 213
								AVWGANFADTQNADLLGHGTHVAGTVGGK	214 – 242
								SAALSVINQGFTWALNDYISK	262 – 282
								GVLNFSGGGPK	289 – 299
								SASQDALWSR	300 – 309
								ATQEGLLVAIAAGNDAVDACNDSPGNIGGSTSGIITVGSIDSSDK	310 – 354
		Q6C080	YALI0_F27071g	Aspartyl protease		*Yarrowia lipolytica* CLIB122	22	YDSAASSSYK	125 – 134
								FDGILGLAYDTISVNK	186 – 201
								IVPPVYNAVNR	202 – 212
								NQFSFFLGDTNK	218 – 229
								GTDGGVATFGGVDEDYFEGK	230 – 249
								ITWLPVR	250 – 256
								GWSGQYTVECDK	310 – 321
		Q6CFP5	YALI0_B05104g	Peptide hydrolase		*Yarrowia lipolytica* CLIB122	21	SNLAEFTDFFTR	130 – 141
								QVNQLAHGYK	158 – 167
								KPTVEYFAHPWGQPSIIAR	172 – 190
								QYFTETK	272 – 278
								YGYPSSFVIESGFQYTSK	349 – 366
								LSFEHMR+Oxidation (M)	378 – 384
								LTIGYAYELAFAK	389 – 401
4.	*Yarrowia osloensis* CBS 10146			Alkaline extracellular protease	65	*Yarrowia sp.* E02	32	AVETTPVTQWGLSR	158 – 171
								ISHKQAQSGNYAYVR	172 – 186
								QAQSGNYAYVR	176 – 186
								ETVGKYPTVAYVVDSGIR	187 – 204
								YPTVAYVVDSGIR	192 – 204
								TTHSEFGGR	205 – 213
								AVWGANFADTTNADLLGHGTHVAGTVGGK	214 – 242
								TYGVDANAK	243 – 251
								TYGVDANAKLVAVK	243 – 256
								LVAVKVFAGR	252 – 261
								SAALSVINQGFTWALNDFISK	262 – 282
								GVLNFSGGGPK	289 – 299
								SASQDALWSR	300 – 309
				Alkaline		*Yarrowia*	26	ETVGKYPTVAYVVDSGIR	187 – 204

				extracellular protease		*sp.*B02		YPTVAYVVDSGIR	192 – 204
								TTHSEFGGR	205 – 213
								AVWGANFADTTNADLLGHGTHVAGTVGGK	214 – 242
								TYGVDANAK	243 – 251
								TYGVDANAKLVAVK	243 – 256
								LVAVKVFAGR	252 – 261
								SAALSVINQGFTWALNDFISK	262 – 282
								GVLNFSGGGPK	289 – 299
								GSPNAVAYNGVGL	442 – 454
5.	*Yarrowia parophonii* CBS 12427			Alkaline extracellular protease	65	*Yarrowia sp.* E02	32	AVETTPVTQWGLSR	158 – 171
								ISHKQAQSGNYAYVR	172 – 186
								QAQSGNYAYVR	176 – 186
								ETVGKYPTVAYVVDSGIR	187 – 204
								YPTVAYVVDSGIR	192 – 204
								TTHSEFGGR	205 – 213
								AVWGANFADTTNADLLGHGTHVAGTVGGK	214 – 242
								TYGVDANAK	243 – 251
								LVAVKVFAGR	252 – 261
								SAALSVINQGFTWALNDFISK	262 – 282
								SAALSVINQGFTWALNDFISKR	262 – 283
								GVLNFSGGGPK	289 – 299
								SASQDALWSR	300 – 309
				Alkaline extracellular protease		*Yarrowia sp.* B02	26	ETVGKYPTVAYVVDSGIR	187 – 204
								YPTVAYVVDSGIR	192 – 204
								TTHSEFGGR	205 – 213
								AVWGANFADTTNADLLGHGTHVAGTVGGK	214 – 242
								TYGVDANAK	243 – 251
								LVAVKVFAGR	252 – 261
								SAALSVINQGFTWALNDFISK	262 – 282
								SAALSVINQGFTWALNDFISKR	262 – 283
								GVLNFSGGGPK	289 – 299
								GSPNAVAYNGVGL	442 – 454
6.	*Yarrowia porcina* CBS 12935	A0A371CCQ4	B0I71DRAFT_162965	Carboxypeptidase	30	*Yarrowia lipolytica* YB392	25	AKDPSSLGLDK	75 – 85
								DPSSLGLDK	77 – 85
								QYSGYLDVEDEDK	88 – 100

								QYDEYEPMACGK	254 – 265
								GGAPPVLDEPTCENMR	266 – 281
								KECDSGTSLCYK	330 – 341
								ECDSGTSLCYK	331 – 341
								DFICNWLGNK	409 – 418
								WTDELEWFGK	420 – 429
								ELSDWVVDGK	436 – 445
								VYEAGHMVPYDQPK	461 – 474
								NSLEMLNSWLAK	475 – 486
								DYSYGSK	487 – 493
				Alkaline extracellular protease		*Yarrowia sp.* B02	20	YPTVAYVVDSGIR	192 – 204
								AVWGANFADTTNADLLGHGTHVAGTVGGK	214 – 242
								TYGVDANAK	243 – 251
								SAALSVINQGFTWALNDFISK	262 – 282
								GVLNFSGGGPK	289 – 299
								SASQDALWAR	300 – 309
				Alkaline extracellular protease		*Yarrowia sp.* E02	20	YPTVAYVVDSGIR	192 – 204
								AVWGANFADTTNADLLGHGTHVAGTVGGK	214 – 242
								TYGVDANAK	243 – 251
								SAALSVINQGFTWALNDFISK	262 – 282
								GVLNFSGGGPK	289 – 299
								SASQDALWAR	300 – 309
		P09230	YALI0F31889g (XPR2)	Alkaline extracellular protease (AEP)		*Yarrowia lipolytica* CLIB122	21	AIQTTPVTQWGLSR	158 – 171
								HPTVSYVVDSGIR	192 – 204
								AVWGANFADTQNADLLGHGTHVAGTVGGK	214 – 242
								SAALSVINQGFTWALNDYISK	262 – 282
								GVLNFSGGGPK	289 – 299
								SASQDALWSR	300 – 309
7.	*Yarrowia alimentaria* CBS 10151	P09230	YALI0F31889g (XPR2)	Alkaline extracellular protease (AEP)	130	*Yarrowia lipolytica* CLIB122	42	AIQTTPVTQWGLSR	158 – 171
								KAQTGNYAYVR	176 – 186
								AQTGNYAYVR	177 – 186
								ETVGKHPTVSYVVDSGIR	187 – 204
								HPTVSYVVDSGIR	192 – 204
								TTHSEFGGR	205 – 213
								AVWGANFADTQNADLLGHGTHVAGTVGGK	214 – 242

								TYGVDANTK	243 – 251
								TYGVDANTKLVAVK	243 – 256
								SAALSVINQGFTWALNDYISK	262 – 282
								GVLNFSGGGPK	289 – 299
								GVLNFSGGGPKSASQDALWSR	289 – 309
								SASQDALWSR	300 – 309
								ATQEGLLVAIAAGNDAVDACNDSPGNIGGSTSGIITVGSIDSSDK	310 – 354
								GSPNAVAYNGVGI	442 – 454
8.	*Yarrowia alimentaria* CBS 10151			Alkaline extracellular protease	110	*Yarrowia sp.* E02	29	ISHKQAQSGNYAYVR	172 – 186
								QAQSGNYAYVR	176 – 186
								ETVGKYPTVAYVVDSGIR	187 – 204
								YPTVAYVVDSGIR	192 – 204
								TTHSEFGGR	205 – 213
								AVWGANFADTTNADLLGHGTHVAGTVGGK	214 – 242
								TYGVDANAK	243 – 251
								TYGVDANAKLVAVK	243 – 256
								LVAVKVFAGR	252 – 261
								SAALSVINQGFTWALNDFISK	262 – 282
								GVLNFSGGGPK	289 – 299
								SASQDALWSR	300 – 309
				Alkaline extracellular protease		*Yarrowia sp.* B02	28	ETVGKYPTVAYVVDSGIR	187 – 204
								YPTVAYVVDSGIR	192 – 204
								TTHSEFGGR	205 – 213
								AVWGANFADTTNADLLGHGTHVAGTVGGK	214 – 242
								TYGVDANAK	243 – 251
								TYGVDANAKLVAVK	243 – 256
								LVAVKVFAGR	252 – 261
								SAALSVINQGFTWALNDFISK	262 – 282
								GVLNFSGGGPK	289 – 299
								SASQDALWAR	300 – 309
								GSPNAVAYNGVGL	442 – 454
		P09230	YALI0F31889g (XPR2)	Alkaline extracellular protease (AEP)		*Yarrowia lipolytica* CLIB122	28	AIQTTPVTQWGLSR	158 – 171
								HPTVSYVVDSGIR	192 – 204
								TTHSEFGGR	205 – 213
								AVWGANFADTQNADLLGHGTHVAGTVGGK	214 – 242

								LVAVKVFAGR	252 – 261
								SAALSVINQGFTWALNDYISK	262 – 282
								GVLNFSGGGPK	289 – 299
								SASQDALWSR	300 – 309
								GSPNAVAYNGVGI	442 – 454
9.	*Yarrowia galli* CBS 9722			Alkaline extracellular protease	40	*Yarrowia sp.* E02	17	ETVGKYPTVAYVVDSGIR	187 – 204
								YPTVAYVVDSGIR	192 – 204
								TTHSEFGGR	205 – 213
								TYGVDANAK	243 – 251
								SAALSVINQGFTWALNDFISK	262 – 282
								SAALSVINQGFTWALNDFISKR	262 – 283
								GVLNFSGGGPK	289 – 299
								GVLNFSGGGPKSASQDALWSR	289 – 309
								SASQDALWSR	300 – 309
				Alkaline extracellular protease		*Yarrowia sp.* B02	18	ETVGKYPTVAYVVDSGIR	187 – 204
								YPTVAYVVDSGIR	192 – 204
								TTHSEFGGR	205 – 213
								TYGVDANAK	243 – 251
								SAALSVINQGFTWALNDFISK	262 – 282
								SAALSVINQGFTWALNDFISKR	262 – 283
								GVLNFSGGGPK	289 – 299
								GSPNAVAYNGVGL	442 – 454
10.	*Yarrowia galli* CBS 9722			Alkaline extracellular protease	37-39	*Yarrowia sp.* B02	22	TTPVTQWGLSR	161 – 171
								ETVGKYPTVAYVVDSGIR	187 – 204
								YPTVAYVVDSGIR	192 – 204
								TTHSEFGGR	205 – 213
								TYGVDANAK	243 – 251
								TYGVDANAKLVAVK	243 – 256
								LVAVKVFAGR	252 – 261
								SAALSVINQGFTWALNDFISK	262 – 282
								GVLNFSGGGPK	289 – 299
								GSPNAVAYNGVGL	442 – 454
				Alkaline extracellular protease		*Yarrowia sp.* E02	19	ETVGKYPTVAYVVDSGIR	187 – 204
								YPTVAYVVDSGIR	192 – 204
								TTHSEFGGR	205 – 213

								TYGVDANAK	243 – 251
								TYGVDANAKLVAVK	243 – 256
								LVAVKVFAGR	252 – 261
								SAALSVINQGFTWALNDFISK	262 – 282
								GVLNFSGGGPK	289 – 299
								GVLNFSGGGPK	300 – 309
		P09230	YALI0F31889g (XPR2)	Alkaline extracellular protease (AEP)		*Yarrowia lipolytica* CLIB122	19	AIQTTPVTQWGLSR	158 – 171
								TTHSEFGGR	205 – 213
								LVAVKVFAGR	252 – 261
								SAALSVINQGFTWALNDYISK	262 – 282
								GVLNFSGGGPK	289 – 299
								SASQDALWSR	300 – 309
								GSPNAVAYNGVGI	442 – 454
11.	*Yarrowia brassicae* CBS 15225	P09230	YALI0F31889g (XPR2)	Alkaline extracellular protease (AEP)	180	*Yarrowia lipolytica* CLIB122	16	AIQTTPVTQWGLSR	158 – 171
								AQTGNYAYVR	177 – 186
								HPTVSYVVDSGIR	192 – 204
								TTHSEFGGR	205 – 213
								TYGVDANTK	243 – 251
								GVLNFSGGGPK	289 – 299
								SASQDALWSR	300 – 309
				Alkaline extracellular protease		*Yarrowia sp.* B02	16	YPTVAYVVDSGIR	192 – 204
								TTHSEFGGR	205 – 213
								TYGVDANAK	243 – 251
								SAALSVINQGFTWALNDFISK	262 – 282
								GVLNFSGGGPK	289 – 299
								SASQDALWAR	300 – 309
